# Alignment-Annotator web server: rendering and annotating sequence alignments

**DOI:** 10.1093/nar/gku400

**Published:** 2014-05-09

**Authors:** Christoph Gille, Michael Fähling, Birgit Weyand, Thomas Wieland, Andreas Gille

**Affiliations:** 1 Department of Biochemistry, Charité, University Medicine Berlin, Charitéplatz 1, 10117 Berlin, Germany; 2 Institute of Vegetative Physiology, Charité, University Medicine Berlin, Charitéplatz 1, 10117 Berlin, Germany; 3 Department of Plastic, Hand and Reconstructive Surgery, Hannover Medical School, Carl-Neubergstraße 1, 30625 Hannover, Germany; 4 Mannheim Medical Faculty, Institute of Experimental and Clinical Pharmacology and Toxicology, Heidelberg University, Maybachstraße 14, 68169 Mannheim, Germany

## Abstract

Alignment-Annotator is a novel web service designed to generate interactive views of annotated nucleotide and amino acid sequence alignments (i) *de novo* and (ii) embedded in other software. All computations are performed at server side. Interactivity is implemented in HTML5, a language native to web browsers. The alignment is initially displayed using default settings and can be modified with the graphical user interfaces. For example, individual sequences can be reordered or deleted using drag and drop, amino acid color code schemes can be applied and annotations can be added. Annotations can be made manually or imported (BioDAS servers, the UniProt, the Catalytic Site Atlas and the PDB). Some edits take immediate effect while others require server interaction and may take a few seconds to execute. The final alignment document can be downloaded as a zip-archive containing the HTML files. Because of the use of HTML the resulting interactive alignment can be viewed on any platform including Windows, Mac OS X, Linux, Android and iOS in any standard web browser. Importantly, no plugins nor Java are required and therefore Alignment-Anotator represents the first interactive browser-based alignment visualization. **Availability:** http://www.bioinformatics.org/strap/aa/ and http://strap.charite.de/aa/.

## INTRODUCTION

Sequence alignment visualization is a widely used method to indicate differences and similarities in bio-polymers and to elucidate structurally and functionally important sequence positions. There already exist multiple web services that utilize or provide sequence alignments. Many of these applications offer their own implementation for server side alignment rendering, e.g. UniProt (1), Pfam (2), SCOPe (3) and T-Coffee (4). This is surprising because software already exists for this task, e.g. ALSCRIPT (5), BoxShade, ESPript (6) and TexShade (7). These programs generate high quality figures as vector graphics e.g. PostScript, PDF and SVG which can be displayed and printed on all computer platforms in high quality. However, vector images are not commonly supported by web browsers without additional plugins. A further and more important shortcoming is that these documents lack interactivity. Interactive capabilities including hyper-links, balloon messages and the option to shield information are becoming increasingly important in the -omics era. Java applets have overcome these shortcomings and have brought real-time interactivity and 3D visualization for alignments (8–10). However, Java constitutes an additional layer of software and thereby carries an own set of technical problems and risks. A not in-frequent problem is that Java applets simply fail to run. In addition there are risks of (i) security threats, (ii) browser crashes and (iii) excessive memory consumption. For this reason, HTML5 has been developed as the new standard for web browsers to natively support multimedia and graphical content. Interestingly, in contrast to the client side, there is the trend of Java usage on the server side. In following these trends, we have designed Alignment-Annotator as a web service to create interactive multiple decorated sequence alignments in HTML5.

## ALGORITHMS AND SOFTWARE

### Implementation

Alignment-Annotator has been implemented in PHP, which uses the Java program Alignment-To-HTML to generate the HTML code (11). The client side program logic is implemented in HTML5 and JavaScript. The interlinked 3D-visualization requires client side Java. The program code to setup an independent server can be requested from the authors.

### Performance tuning

A major challenge has been the computational time requirement for the generation of the alignment on the server side. Depending on the selected options, significant computations and external data retrieval may be involved. Therefore, we have rigorously optimized processing speed by the following strategies:

- Identification of database IDs and homologous structures by the fast similarity/identity search program Blat (see below for more details).
- Caching of downloaded data and computational results.
- Holding of copies of the UniProtKB, the UniProt annotations, the PDB and the Catalytic Site Atlas on the server.
- Load balancing and process prioritization. This allows for termination of time consuming computations in favor of recently submitted jobs and the option to resume terminated jobs at a later time.
- Parallelization of data retrieval and computations.
- Collective item requests from remote services.

The performance tuning was essential for usability and has significantly increased processing speed by 10-fold compared to the initial implementation.

### Alignment computation

Submitted sequences undergo alignment computation unless the presence of gaps indicate that sequences are already aligned. By default, a rapid alignment method is used (12), whereas more accurate and time demanding methods like T-Coffee (4) are available.

A total of eight different sequence alignment methods and five structure alignment programs can be specified in the first script, which is executed before alignment computation. Sequence based methods are usually sufficient if sequences are not too dissimilar. Mixed sequence/3D structure alignment can improve alignment quality of remote homologs and is provided as an option. However, 3D structure alignment requires more time, depending on the number and size of the 3D structures.

### Assignment of database IDs

The UniProt IDs of all input sequences are determined by perfect sequence match unless the sequence ID is explicitly specified in the input files or by script commands.

We utilize Blat, which is about 50 times faster than Blast. It is optimized for databases that are small enough to fit into the main memory (13). It is used for similarity search in the PDB and for exact-match search in UniProtKB. For the latter we were able to reduce computation time and the required amount of memory (RAM) from current 192 to only 54 MB. We achieved this by running Blat in nucleotide mode using a degenerated amino acid alphabet.

The database identifiers are used to interrogate annotation servers. BioDAS is regarded as a standard for the exchange of residue specific information using XML (14,15). The advantage of this standard is that the same program code fits all different services. The available services are listed in the BioDAS-registry file, which is updated regularly in Alignment-Annotator. Thereby future services will be automatically available in Alignment-Annotator.

### Visualization of annotations

A simple and widely used method to display sequence annotations is to change the residue's foreground or background color. However, this does not allow discrimination of overlapping residue selections. This presents a real limitation as more and more sequence features are discovered and need annotation. Alignment-Annotator emphasizes residues by underlining. Text attributes and hyperlinks are exhibited in the balloon messages. This approach is more versatile and allows for multiple overlapping annotations. The layout of the residue selections is optimized for compactness. In order to avoid duplication of annotations, the server maps all possible annotation names to a controlled dictionary. Thereby equivalent annotations using different vocabulary e.g. Phosphoserine and Phosphorylated_serine are recognized and are merged. If an annotation constitutes a generalization of another one, the less precise is discarded in favor of the more precise annotation. For example, for the three annotations at the same sequence position, Phosphotyrosine, Modified_Residue and Post_translational_modification, only Phosphotyrosine is retained because it is most precise. In addition, the program performs a consistency check on all modified amino acids. For example, Phosphotyrosine has to be the letter Y.

## RESULTS

### Embedding in other software

**Figure 1.**
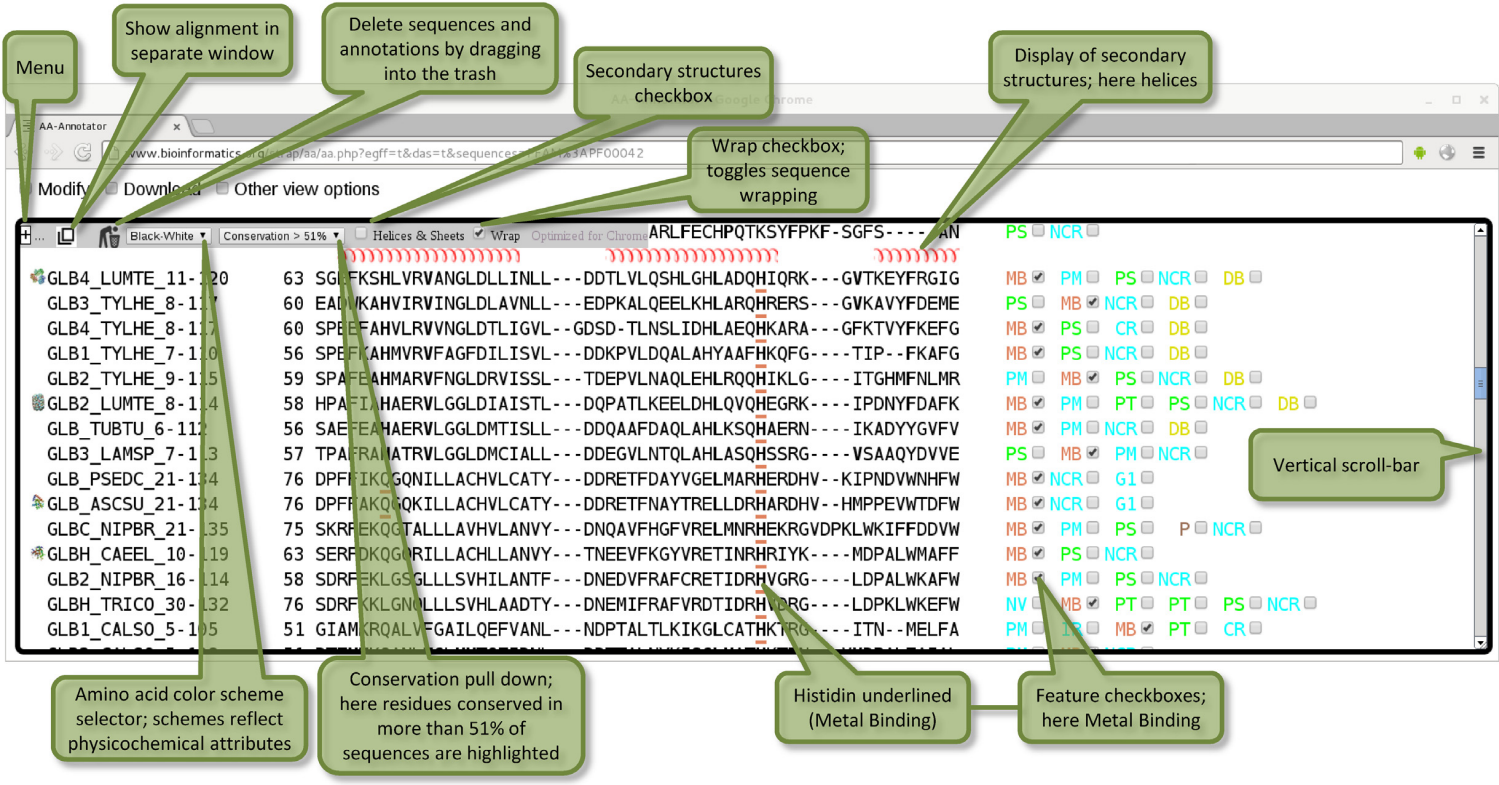
Pfam globin family PF00042 in Alignment-Annotator opened with the address: http://www.bioinformatics.org/strap/aa/aa.php?egff=t&das=t&sequences=PFAM%3APF00042. The program parameters embedded in this URL are egff = t, das = t, sequences = PFAM:PF00042. The page consists of two parts: (i) A user interface at the top for interaction with the server. (ii) The scrollable alignment document surrounded by a black border. The trailing check-boxes on the right allow to activate annotations in individual and all rows. Currently ‘Metal-Binding’ is selected. Each alignment block is headed by a secondary structure cartoon.

Alignment-Annotator is the first browser-based interactive alignment viewer, which runs on all platforms. Other programs can use Alignment-Annotator for visualization of dynamically generated alignments. Input data or a reference to the input data is submittable within the URL. For example, the browser page in Figure 1 is obtained with a URL containing PFAM:PF00042 which denotes the database name and the Pfam ID of the globin family (2). In addition, residue annotations, balloon messages and icons can be transmitted.

### Annotations

Alignment-Annotator allows user-defined residue annotations. For amino acid sequences, it displays annotations obtained from services and can derive information from PDB structure files. Data sources include several BioDAS services, the Catalytic Site Atlas (16) and the UniProt. Alignment-Annotator supports large numbers of overlapping annotations. However, most annotations obtained automatically may not be of interest to the user, and can be temporarily or permanently removed.

### 3D-structures

When this option is activated, the most similar PDB entry of each sequence is used to obtain further residue specific information recorded in the PDB file. This is the secondary structure type such as α-helix, β-sheet, the residue number and chain letter and post-translational modification such as S-acetylation of cysteine. The secondary structure of one sequence in the alignment is displayed as a cartoon at the top. This is usually the one where the secondary structure spans the longest range. For 3D structures containing ligands, e.g*.* heme in hemoglobin, residues in spatial neighborhood can be highlighted. Each sequence with a mapped 3D structure has a button to display that structure through the OpenAstex 3D-viewer. The default 3D-rendering style can be customized with the Strap scripting language in an implementation independent fashion. This retains the option to implement further 3D software in the future. The 3D style of selected amino acids can be changed by attaching 3D commands to residue annotations. Furthermore, several structures can be displayed together in one view and superimposed to compare their structures. Homologous PDB entries enable other knowledge data bases: The Catalytic Site Atlas is a manually curated collection of active site triads.

## DISCUSSION

Alignment-Annotator derives information from similar PDB structures even if the amino acid sequence is not identical. The information recorded in the PDB file or the Catalytic Site Atlas may not be correct for the sequence under investigation. This is because functional features of proteins like catalytic activity or ligand binding change during evolution. A well known text book examples are proteins of the eye lens, the crystallins, which are derived from enzymes but have lost catalytic activity. To emphasize potentially incorrect information, these annotations are labeled ‘By similarity’ as opposed to ‘By identity’ or the default evidence level. Annotations with adjacent sequence mismatches between the sequence and the 3D-structure are filtered out.

### Desktop application versus online tool

Versatile commercial or free software for alignments exist (CLC-Workbench, Strap (17) and Jalview (18)), which require training but exhibit a large degree of flexibility. In contrast, Alignment-Annotator was designed as a web service with an easy-to-use graphical user interface which does not require local installation and can be embedded in other applications. Both approaches have pros and cons summarized in Table 1.

**Table 1.** Comparison of desktop applications and Alignment-Annotator

	Desktop application	Alignment-Annotator
Installation effort	Configuration of Java and web-proxy	None
Adding sequences	Drag and drop of files and accession IDs	Copy and paste
Functionality	Comprehensive	Essential
Usability	Highly complex	Simple
Responsiveness	Rapid	Some lag time
Security	Local files are accessed	Safe operation in sandbox
Maximum alignment size	Very large	Medium

## CONCLUSION

Alignment-Annotator is an alignment visualization platform for web services and for creating alignment views *de novo*.
